# Neuroependymal denudation is still in progress in full-term human fetal spina bifida aperta

**DOI:** 10.1186/1743-8454-7-S1-S19

**Published:** 2010-12-15

**Authors:** Deborah Sival, Montserrat Guerra, Wilfred den Dunnen, Frederico Bátiz, Genaro Alvial, Esteban Rodríguez

**Affiliations:** 1 Department of Pediatric Neurology University Medical Center Groningen, University of Groningen, Beatrix Children's Hospital, Hanzeplein 1, PO Box 30001, 9700RB, Groningen, The Netherlands; 2 Instituto de Anatomía, Histología y Patología, Facultad de Medicina, Universidad Austral de Chile, Valdivia, Chile; 3 Department of Pathology and Medical Biology, University Medical Center Groningen, University of Groningen, Beatrix Children's Hospital, Hanzeplein 1, PO Box 30001, 9700RB, Groningen, The Netherlands

## Background

Background: In human spina bifida aperta (SBA), cerebral pathogenesis (hydrocephalus, Sylvian aqueduct (SA) stenosis and heterotopias) is poorly understood. In animal models (such as hyh mutant mice) the loss of the ventricular lining (ependymal denudation) causes SA stenosis and hydrocephalus. In these animals, ependymal denudation is ascribed to an alteration in junction proteins. Analogous to studies in laboratory animals, we aimed to investigate ependymal denudation in human fetal SBA.

## Materials and methods

Sections through SA of five SBA and five control fetuses (median gestational ages 37 and 40 weeks, respectively) were immunostained for markers of ependyma (caveolin1, βIV-tubulin, S100), blood vessels (Glut-1), astrocytes (GFAP) and junction proteins (N-cadherin, connexin-43, neural cell adhesion molecule (NCAM)).

## Results

In all five control fetuses, ependymal denudation was absent. In all five SBA fetuses, different stages of ependymal denudation were concurrently observed, consisting of: (I) intact ependyma/neuroepithelium; (II) imminent ependymal denudation (with abnormal sub-cellular location of junction proteins in cytoplasm instead of at the plasma membrane); (III) ongoing ependymal denudation (with protrusion of neuropil into SA, formation of rosettes and macrophage invasion); and (IV) completed ependymal denudation (with astroglial reaction).

## Conclusions

In full-term SBA fetuses, intra-individual concurrence of imminent, ongoing and completed ependymal denudation implicates that ependymal denudation would continue after birth. At the areas associated with imminent ependymal denudation, the abnormal expression of junction proteins suggests that abnormal formation of gap and adherent junctions precedes defective ependymal coupling, desynchronized ciliary beating, ependymal denudation and hydrocephalus.

